# Immune and Apoptosis Mechanisms Regulating Placental Development and Vascularization in Preeclampsia

**DOI:** 10.3389/fphys.2020.00098

**Published:** 2020-02-11

**Authors:** Nozha Raguema, Sarah Moustadraf, Mariane Bertagnolli

**Affiliations:** Laboratory of Maternal-Child Health, Centre de Recherche de l’Hôpital du Sacré-Coeur de Montréal, Centre Intégré Universitaire de Santé et de Services Sociaux du Nord-de-l’Île-de-Montréal, Montreal, QC, Canada

**Keywords:** apoptosis, preeclampsia, placental development, vascular remodeling, hypoxia

## Abstract

Preeclampsia is the most severe type of hypertensive disorder of pregnancy, affecting one in 10 pregnancies worldwide and increasing significantly maternal and neonatal morbidity and mortality. Women developing preeclampsia display an array of symptoms encompassing uncontrolled hypertension and proteinuria, with neurological symptoms including seizures at the end of pregnancy. The main causes of preeclampsia are still unknown. However, abnormal placentation and placenta vascularization seem to be common features in preeclampsia, also leading to fetal growth restriction mainly due to reduced placental blood flow and chronic hypoxia. An over activation of maternal immunity cells against the trophoblasts, the main cells forming the placenta, has been recently shown as an important mechanism triggering trophoblast apoptosis and death. This response will further disrupt the remodeling of maternal uterine arteries, in a first stage, and the formation of new placental vessels in a later stage. A consequent chronic hypoxia stress will further contribute to increase placental stress and exacerbate systemic circulatory changes in the mother. The molecular mechanisms driving these processes of apoptosis and anti-angiogenesis are also not well-understood. In this review, we group main evidences suggesting potential targets and molecules that should be better investigated in preeclampsia. This knowledge will contribute to improve therapies targeting a better placenta formation, having a positive impact on maternal disease prevention and on fetal development.

## Introduction

Pregnancy is a major physiologic event in a woman’s life, leading to significant body and metabolic changes. During the implantation of the embryo, a new transient organ is formed connecting mother and fetus, the placenta (Burton and Fowden, 2015). The structure of the placenta will, during pregnancy, provide a range of functions such as fetal nutrition and oxygenation, as well as the secretion of endocrine factors, also building a maternal-fetal immune tolerance (Heerema-McKenney, 2018).

During the first three gestational weeks, the blastocyst implants in the decidual endometrium, developing the placenta. The implantation is mediated by the invasion of trophoblasts, the main cells forming the placenta (Knofler et al., 2019). Trophoblasts are primitive cells giving origin to different cell lineages such as the cytotrophoblasts and the syncytiotrophoblasts. More specifically, during invasion, the cytotrophoblasts form a cell layer under the syncytiotrophoblasts, so these can infiltrate in between uterine epithelial cells in order to ensure the implantation of the embryo (Knofler et al., 2019).

During the implantation process, endometrial cells, and uterine vessels, in addition to uterine immune cells, are modified to form the decidua. Trophoblasts will, in addition, invade the spiral arteries of the uterus. In order to reach this goal, trophoblasts differentiate into villous trophoblasts, ensuring the feto-maternal exchanges and the endocrine functions of the placenta, and in invasive extravillous trophoblasts, essential for the implantation and remodeling of the uterine vessels. The extravillous trophoblast proliferates and becomes invasive, migrating into the decidua and myometrium.

The migration of the extravillous trophoblasts into the maternal spiral arteries also represents another key step of human placentation (Baker et al., 2009). There, extravillous trophoblasts will replace maternal arteries endothelium and smooth muscle cells by stimulating their apoptosis and ultimately promote vessel remodeling (Whitley and Cartwright, 2009). The invasion of extravillous trophoblasts further reduces vasomotor tonus and responses of spiral arteries to vasoactive factors (Baker et al., 2009), causing a permanent arterial dilation and ensuring an increased blood flow to the placenta and growing fetus (Salamonsen et al., 2003). This remodeling process is completed around 20 gestational weeks in healthy pregnancies (Osol and Mandala, 2009).

However, some pregnancies are associated with complications. Indeed, several studies have demonstrated defects in the vascular remodeling process in more severe complications such as preeclampsia (da Cunha Castro and Popek, 2018). In abnormal placentation with poor vascularization of the placenta, the transition of increased oxygenation does not occur, resulting in tissue hypoxia. In addition, the failure of vascular remodeling can reduce significantly the blood supply to the placenta, stimulating a placental metabolic stress and the release of vasoactive factors. As a result, maternal blood pressure can increase significantly either directly due to the chronic rise in uterine vascular resistance, or in response to placental vasoactive factors, causing hypertension in the mother (Karthikeyan and Lip, 2011; American College of Obstetricians and Gynecologists, and Task Force on Hypertension in Pregnancy, 2013; Chaiworapongsa et al., 2014; Dymara-Konopka et al., 2018). Indeed, hypertension occurs in 10% of all pregnancies in the world. If it is not treated or is not well-monitored during pregnancy, it can predispose to more severe outcomes such as preterm delivery, low birth weight and preeclampsia.

In this review, we will describe one of the most severe hypertensive disorders of pregnancy, preeclampsia. We will discuss how the molecular mechanisms linked to abnormal placentation involving maternal inflammation by activation of immune cells and apoptosis can alter placental vascularization and contribute to the unfavorable progression of preeclampsia.

## Pathogenesis of Preeclampsia

Preeclampsia is a disorder emerging around 20 gestational weeks; however, it is diagnosed through high blood pressure values (≥140/90 mmHg) and proteinuria of ≥ 0.3 g per day later during gestation. Other symptoms can be also associated such as edema, severe headaches and visual disturbance (Ben Ali Gannoun et al., 2016). Considered a major cause of maternal and fetal mortality and morbidity, preeclampsia affects 2 to 10% of all pregnancies worldwide with nearly 70,000 maternal deaths annually (Duhig et al., 2018). It can lead to multi-systemic and serious complications in the mother such as cerebral hemorrhage, renal failure, HELLP syndrome, which is characterized by associated hemolysis and liver injury, and even eclampsia with severe seizures in more severe cases.

In addition, the fetus can also be affected by preeclampsia since it is one of the leading cause of premature birth and intrauterine growth restriction, mainly due to insufficient nutrient supply and chronic hypoxia exposure (Redman and Sargent, 2005). In addition, the only known treatment of preeclampsia to date is premature delivery. Nevertheless, there are risk factors already identified and they include previous history of preeclampsia or family history, multiple pregnancies, as well as cardiometabolic factors such as obesity, diabetes and chronic hypertension (Dong et al., 2017).

The pathogenesis of preeclampsia is still not well-understood and seems to associate differential complex alterations in the placenta and maternal circulation, turning this disorder a major subject of a large amount of studies. Some, however, agree that the preeclampsia origin is an abnormal placentation, which can be defined by two distinct developmental stages: the pre-clinical and the clinical stages (Mayrink et al., 2018). While the pre-clinical stage is determined by critical changes in placental structure and development, the clinical stage is characterized by circulatory systemic changes and symptoms in the mother.

The pre-clinical stage corresponds to inefficient trophoblast invasion in the maternal decidua (Tsatsaris et al., 2008). If the cell invasion occurs only superficially at this stage, trophoblasts will fail to reach the maternal spiral arteries and to promote the vascular remodeling, resulting in increased arterial resistance and reduced blood flow (Roberts and Hubel, 2009). A disrupted remodeling of maternal spiral arteries is, therefore, a major consequence of the pre-clinical stage. It further contributes to promote placental ischemia and to stimulate the secretion of pro-inflammatory factors by the placenta, leading to the second stage of abnormal placentation (Mayrink et al., 2018). Following the pre-clinical stage, the clinical stage corresponds to the manifestation of symptoms in the mother such as severe, and sometimes uncontrolled, high blood pressure, and proteinuria followed by the dysfunction of multiple organs (Chaiworapongsa et al., 2014).

Therefore, it is now well-understood that a healthy placentation depends on the essential steps of placental development and the ability to respond or adapt to different types of stress. The dysfunction of one of these processes can have harmful repercussions on maternal circulation and fetal development.

In the pre-clinical and clinical stages, the maternal immune cells are also active. Lymphocyte T cells can significantly increase the production of inflammatory cytokines such as tumor necrosis factor alpha (TNFα) and interleukin-6 (IL6), as shown in preeclampsia. Pro-inflammatory responses are also associated with a reduction in anti-inflammatory cytokines (de Oliveira et al., 2010; Hutabarat et al., 2017). In maternal vessels, an increase in pro-inflammatory TNFα and IL6 contributes to causing endothelial dysfunction, which is a hallmark of preeclampsia mainly characterized by reduced production of vasodilation factors and an increase in the permeability of endothelial cells (de Oliveira et al., 2010). TNFα is also shown to decrease nitric oxide synthase (NOS) gene transcription while increasing the production of the potent vasoconstrictor endothelin-1 (Cornelius, 2018). Further, placental ischemia can trigger an oxidative stress state, characterized by the bursting of large quantities of reactive oxygen species (ROS) in the cell membrane, the endoplasmic reticulum and the compartments of the mitochondria, causing protein and DNA damage (Schoots et al., 2018). Hence, all these mechanisms contribute to stimulate a state of chronic inflammation during abnormal placentation.

Inflammation can further activate and promote the programed death of trophoblasts, a process called apoptosis. In normal healthy pregnancies, the mechanisms of apoptosis can be of crucial importance to protect the trophoblasts from the attack of maternal immune cells and to promote the death of uterine arteries endothelial cells and their replacement by extravillous trophoblasts (Abrahams et al., 2004; Ashton et al., 2005). However, under inflammation, activation of apoptosis can backfire on the trophoblasts, significantly disrupting trophoblast migration and placental vascularization, also exacerbating immune responses (Sharp et al., 2010).

In addition, it has been described that abnormal placentation can be associated with an imbalance in the production of pro-angiogenic and antiangiogenic factors by the placenta. Increased secretion of antiangiogenic factors soluble fms-like tyrosine kinase 1 (sFlt1) and soluble endoglin (sEng), as well as reduced production of pro-angiogenic factors vascular endothelial growth factor (VEGF) and placental growth factor (PlGF) during exposure to ischemia, likely contributes to the pathogenesis of preeclampsia (Karthikeyan et al., 2011; Helmo et al., 2018). An increasing number of studies have identified enhanced sFlt1 expression in placentas of pregnancies with preeclampsia (Helmo et al., 2018). sFlt1 is the soluble form of VEGF receptor 1 (VEGFR1) and antagonizes the pro-angiogenic effects of VEGF. In addition, sEng, another key antiangiogenic protein, is also shown to be up-regulated in preeclampsia (Dymara-Konopka et al., 2018). On the other hand, the synthesis of pro-angiogenic PlGF was associated with an exacerbated production of anti-angiogenic factors during preeclampsia (Karthikeyan et al., 2011). These results indicate that abnormal placentation does not only cause changes in uterine vessels but also a systemic damage to the maternal endothelium and the angiogenesis capacity, which can lead to endothelial dysfunction (Eddy et al., 2018).

In the next sessions, we describe how these two interconnected mechanisms involving the maternal immune system and cell death act during placentation and placental vascularization in healthy pregnancies and in the pathology of preeclampsia.

## Immune System in Preeclampsia

Several hypotheses have been proposed to explain an abnormal trophoblast invasion and the over activation of placental inflammation in early pregnancies with preeclampsia. Some support an impaired maternal immune response or a defective maternal immune-tolerance to the semi-allogeneic fetus (Racicot et al., 2014). A successful invasion in normal pregnancies relies on an adequate interaction between trophoblast cells and maternal epithelial, immune and endothelial cells and tissues (Liu et al., 2017). In this regard, the maternal immune system plays a key role in facilitating the interaction of two immunologically different beings, the mother and the fetus (Baker et al., 2009).

In normal pregnancies, the processes of trophoblast invasion and spiral artery remodeling are highly dependent on the maternal immune system to allow significant tissue changes (Faas and de Vos, 2017a). During trophoblast invasion, the decidua contains a high number of immune cells necessary for the migration of trophoblasts (Racicot et al., 2014). They include macrophages, natural killer cells (NK), dendritic cells (DCs), T lymphocyte and T regulatory cells (Tregs) (Liu et al., 2017). These cells infiltrate the decidua and gather around the trophoblasts allowing them to reach the endometrium and spiral arteries (Figueiredo and Schumacher, 2016). On the other hand, Tregs and regulatory cytokines ensure the proper control and function of pro-inflammatory cells and their actions during invasion (Baker et al., 2009). In mouse models, it is well-documented that Treg cells are important for maternal-fetal immune tolerance (Faas and de Vos, 2017a). In addition, it is believed that the DCs present in the decidua can promote a dominant presence of T helper type 2 cells (Th2) in the uterus and placenta in order to induce immune tolerance of mother to fetus (Figueiredo and Schumacher, 2016; Cornelius, 2018).

However, an over activated immune system can play a significant negative role in placental development and the progression of preeclampsia. Epidemiological evidence supports this role, indicating an association between adverse changes in maternal immune responses and preeclampsia (Kestlerova et al., 2012). In particular, the fact that preeclampsia can surge more frequently in the first pregnancy, and also that long-term exposure to partner’s sperm has been described as reducing the risk of preeclampsia, or that risks may increase during artificial reproduction, for example, support the hypothesis that the immune system can have a direct impact and perhaps even induce preeclampsia (Matthiesen et al., 2005). In addition, the mechanisms allowing the semi-allogenic trophoblast cell to invade maternal tissues by outwitting maternal processes of non-self recognition can also fail during preeclampsia (Cornelius, 2018).

One hypothesis is that over activation of immune cells stimulates trophoblast apoptosis in preeclampsia (Jafri and Ormiston, 2017). Indeed, during healthy pregnancies, the non-recognition of trophoblasts by maternal immune cells reduces the activation of inflammation, and this will consequently decrease the lysis of the trophoblasts present in the decidua (Cornelius, 2018). In addition, a recent study showed a very low presence of macrophages during trophoblast invasion in normal pregnancies (Taylor and Sasser, 2017). However, in preeclampsia, their numbers can be dramatically increased, notably in the uterine artery wall of patients, where very few extravillous trophoblasts were also present.

T lymphocytes along with NKs and DCs have also been found to respond differently in preeclampsia compared to normal pregnancies, tending to develop a pro-inflammatory state (Faas and de Vos, 2017b). These cells produce and respond to a broad spectrum of cytokines and are involved in paracrine mechanisms regulating trophoblast invasion (Moffett-King, 2002). *In vitro*, maternal macrophages could further induce apoptosis of periarterial extravillous trophoblasts (Miko et al., 2009; Faas and de Vos, 2017b; Taylor and Sasser, 2017). In addition, excessive activation of neutrophils and monocytes in clinical and experimental preeclampsia have also been described (Faas and de Vos, 2017a; Cornelius, 2018). Monocytes have been found to spontaneously synthesize larger quantities of pro-inflammatory cytokines such as IL1b, IL6, and IL8 (Faas and de Vos, 2017a).

Another defective system appears to be involved in the dysfunctional invasion of trophoblasts in the spiral arteries. Human leukocyte antigens (HLAs) are major histocompatibility complex (MHC) molecules expressed on different cells of the placenta. Invasive extravillous trophoblasts express Class I HLA-C and the atypical class Ib antigens HLA-G, which promote immune-regulation between extravillous trophoblasts and maternal decidual NK cells. HLA-G is a ligand for the inhibitory receptor KIR2DL4 of NK cells (Chen et al., 2010). Consequently, the expression of HLA-G by the extravillous trophoblast can defend it against cell death mediated by NKs. This is an essential mechanism of extravillous trophoblast protection to allow cell invasion during healthy pregnancies mainly by inhibiting NK cytotoxicity and cytokine production (Chen et al., 2010; Liu et al., 2013). HLA-G can also directly modify the biological function of trophoblast cells stimulating invasiveness by different cell signaling pathways (Liu et al., 2013). However, a lower HLA-G expression in the placenta, more specifically in extravillous trophoblasts, has been described in preeclampsia (Yie et al., 2004; Robillard et al., 2011).

Lower expression of other HLA genes can inversely protect the placenta. For example, syncytiotrophoblasts do not express HLA-A and HLA-B genes, which leads to not expressing MHC-I, a major binding site for lymphocyte T cells (Thellin and Heinen, 2003; Racicot et al., 2014; Cornelius, 2018). Therefore, by being in close contact with the activated maternal immune system, the lack of HLA-A and -B protects the syncytiotrophoblasts from maternal T cells, hence preventing tissue rejection (Hutabarat et al., 2017).

## Apoptosis in Preeclampsia

**FIGURE 1:**
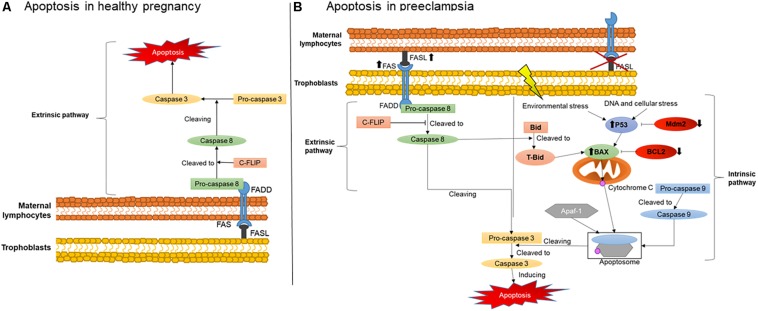
Placental apoptosis mechanisms. **(A)** Extrinsic apoptosis pathway activation in maternal immune cells stimulated by trophoblasts producing FASL. **(B)** Extrinsic apoptosis activation in trophoblasts stimulated by FASL produced by the maternal immune system and intrinsic pathway activated by environmental stress such as hypoxia and DNA damage. FAS, FAS receptor; FASL, FAS ligand; FADD, Fas-associated protein with death domain; C-FLIP, cellular FLICE (FADD-like IL-1β-converting enzyme)-inhibitory protein; P53, apoptosis inductor; BCL-2, B-cell lymphoma 2; BAX, BCL2-associated X apoptosis regulator; Mdm2, Human mouse double minute 2; Bid, BH3 interacting-domain death agonist; tBid, truncated p15 BID; Apaf-1, apoptotic protease activating factor 1.

An important mechanisms involved in maternal-fetal immune tolerance is apoptosis, which is defined as a programed cell death that can be induced by two different pathways: the extrinsic and the intrinsic (Elmore, 2007). The mechanisms of activation of these two pathways are illustrated in Figure 1. The extrinsic pathway is initiated when a cell expressing a FAS receptor on the surface binds to its FAS ligand (FASL), activating a cascade of pro-apoptotic elements and leading to cell death. The binding of FASL to the transmembrane receptor FAS recruits procaspase 8, further activated to caspase 8. This then cleaves another type of procaspase generating the active form of caspase 3. Active caspase 3 will also recruit other downstream effectors, promoting the final steps of cell death (Elmore, 2007).

The intrinsic pathway is usually activated during intracellular stress such as ischemia and DNA damage, or when cells receive signals from certain pro-apoptotic factors. During intracellular stress, P53 is also activated, a protein which main role is to stimulate cascades of pro-apoptotic factors mainly targeting the mitochondria (Harris and Levine, 2005). One of these factors, a pro-apoptotic member of the BCL2 family called Bax, releases cytochrome C from the inner mitochondrial membrane during stress (Sharp et al., 2014). Subsequently, cytochrome C can form an apoptosome in the cytosol, a complex comprising active caspase 9 and Apaf-1, in order to cleave procaspase 3 to its active form and, therefore, cause cell death (Sharp et al., 2010).

Figure 1 also illustrates the critical changes in apoptosis pathways during normal healthy and complicated pregnancies. During healthy and abnormal placentation, the mechanisms of apoptosis, as noted above, occur in the vast majority of placental cells and likewise in placental villous trophoblasts. They regulate the number and type of trophoblast population in the placenta and decidua, greatly influencing maternal-fetal immune tolerance (Sharp et al., 2010). Apoptosis is particularly important in the initial and final stages of placental development and function. Apoptotic cells have been found in the maternal and embryonic parts of the placenta during normal pregnancy. The presence of these cells is associated with stages of placental development, including the invasion of trophoblast, remodeling of the spiral arteries, differentiation of trophoblasts and childbirth (Abrahams et al., 2004). However, stimulation of placental apoptosis is also regulated by maternal immune cells, although the main mechanisms activated by these cells in normal and complicated pregnancies are not yet fully understood.

Evidence supports that in normal pregnancies, trophoblasts secrete FASL rather than expressing the FAS receptor (Kauma et al., 1999), as shown in Figure 1A. The predominance of FASL secretion reduces their susceptibility to activate themselves and enter apoptosis (Bamberger et al., 1997). Therefore, the higher FASL secretion combined with lower FAS receptor expression may protect trophoblasts against cytolytic activation of maternal immune cells. Instead, they can stimulate the extrinsic apoptosis pathway in maternal immune cells such as T lymphocytes and macrophages, naturally expressing higher amounts of FAS receptor (Kauma et al., 1999; Roh et al., 2002). In addition, the higher secretion of FASL by trophoblasts is an important mechanism activating apoptosis of endothelial cells in the maternal spiral arteries (Pongcharoen et al., 2004). This process is therefore critical for their replacement by extravillous trophoblasts during normal placentation (Pongcharoen et al., 2004).

On the other hand, changes in this process and on main players have a significant negative impact on placental development, contributing to the establishment of placental abnormalities. In hypertensive pregnancies, for example, increased trophoblast apoptosis and reduced cell invasion were described (Whitley et al., 2007). More specifically, during preeclampsia, apoptosis was described in extravillous trophoblasts in the vicinity of the wall of the spiral arteries, rather than in maternal endothelial cells (Whitley et al., 2007). It has also been shown that these extravillous trophoblasts, instead of mainly expressing FASL, express FAS receptor and the TNF-α receptor 1, another pro-apoptotic factor. They also had lower expression of anti-apoptotic factors BCL2 and Mcl-1.

Consistent with these findings, we and others have also shown that the expression of FAS and FASL in serum and in maternal lymphocytes also occurs in preeclampsia (Miko et al., 2009; Raguema et al., 2018). We also described the polymorphisms of FAS-670A/G and FASL IVS2nt124A/G genes in the lymphocytes of pregnant women with preeclampsia. These polymorphisms were characterized by a higher production of FASL by the maternal immune cells, supporting the postulate of an over activation of pro-apoptosis responses in the trophoblasts, as illustrated in Figure 1B, which could contribute to alter the placentation during preeclampsia (Abrahams et al., 2004). In line with our findings, another study also described increased expression of the FAS receptor and reduced expression of FASL in the placentas of pregnancies with hypertensive disorders (Roh et al., 2002).

In addition, over activation of the intrinsic pathway may also occur due to reduced anti-apoptotic factors, being another potential origin of exacerbated apoptosis in preeclampsia (Figure 1B) (Hung et al., 2002; Sharp et al., 2010). Indeed, several studies have shown that, during the development of the trophoblastic villi, an early stage of apoptosis is activated, specifically targeting cytotrophoblasts, whereas later stages of apoptosis are more likely to affect syncytiotrophoblasts (Hutabarat et al., 2017). Syncytiotrophoblasts are multinucleated cells with number of nuclei increasing up to nine times from the beginning of gestation to term. These nuclei can form aggregates called syncytial knots. In normal pregnancies, knots are rarely seen before 20 weeks of gestation and their frequency increases in mature placentas (Loukeris et al., 2010). They are also very frequent in the placentas of complicated pregnancies, being present on almost all terminal villi in preeclamptic placentas (Tomas et al., 2011). Currently, the presence of a large number of syncytial knots can indicate premature aging of the placentas (Loukeris et al., 2010).

Syncytiotrophoblast apoptosis in preeclampsia has also been associated with larger numbers of syncytial knots and higher oxidative stress (Tomas et al., 2011; Fogarty et al., 2013). Rajakumar et al. have also described the presence of syncytial knots in the bloodstream of preeclamptic mothers (Rajakumar et al., 2012). In their study, they described the presence of large structures of 50–150 μm consistent with detached syncytial knots after gently rinsing the placentas of preeclamptic pregnancies. The syncytial origin was determined by the fact that these structures were always membrane-bound and multinucleated. Interestingly, in normal term placentas, very few multi-nucleated structures were observed in the effluent. Their data indicated that the abundant syncytial knots in the preeclamptic placentas can easily detach from the syncytial layer to become free aggregates of syncytial origin. Furthermore, these syncytial aggregates have been described as containing a large expression of sFlt1, potentially contributing to impair placental angiogenesis in preeclampsia (Rajakumar et al., 2012).

The increase of syncytial knots in trophoblasts has been observed and associated with down regulation of anti-apoptotic factors of the intrinsic pathway such as BCL2 and Mdm2 and with premature aging of the placenta in preeclampsia (Coleman et al., 2013). In addition, they can also increase significantly when exposed to hyperoxia, hypoxia or greater amounts of ROS in the placental villi (Heazell et al., 2007). These findings indicate that the presence of knots in syncytiotrophoblasts may reflect placental responses to environmental stress and the activation of intrinsic apoptosis pathways in abnormal placentation and preeclampsia.

## Summary

Despite the increasing number of studies reporting the involvement of the maternal immune system and apoptosis in the development of the placenta and preeclampsia, the main mechanisms promoting cell death and ineffective vascular remodeling in abnormal placentation are still unclear. It is well-known that close interaction between the maternal immune system and trophoblasts is essential during placentation. However, in the presence of an over activation of inflammation and apoptosis of the trophoblasts, it leads to abnormal placentation with impaired invasion, vascular remodeling and microcirculation in the placenta. Activation of different pathways of apoptosis also depends on pathological processes in the placenta. More specifically, activation of the FAS/FASL extrinsic pathway and of the intrinsic pathway mediated by P53, Bax, and BCL2 have been systematically reported in normal and complicated pregnancies such as preeclampsia although their mechanisms are not yet fully understood.

Future studies are therefore necessary to better understand the mechanisms of activation of the extrinsic and intrinsic apoptosis pathways in hypertensive disorders of pregnancy and whether their activation may also depend on different pathological stimuli associated with preeclampsia. Current data support that activation of the extrinsic pathway is largely associated with systemic and local immune and inflammatory responses in the placenta. The intrinsic pathway is primarily described as a consequence of trophoblasts disruption under severe and chronic hypoxia, DNA damage and environmental stress conditions. Importantly, the close interaction of these two pathways can help promote abnormal placentation and vascularization, aggravating placental stress during preeclampsia. More studies evaluating these mechanisms are therefore needed to elucidate these interactions and to investigate new therapies capable of reducing the activation of apoptosis at different stages of the development of preeclampsia.

## Author Contributions

NR wrote the manuscript and supervised figures edition. SM designed the figures and collaborated in writing. MB reviewed and edited the text and figures.

## Conflict of Interest

The authors declare that the research was conducted in the absence of any commercial or financial relationships that could be construed as a potential conflict of interest.

## References

[B1] Abrahams V. M. Straszewski-Chavez S. L. Guller S. Mor G. (2004). First trimester trophoblast cells secrete Fas ligand which induces immune cell apoptosis. *Mol. Hum. Reprod.* 10 55–63. 10.1093/molehr/gah006 14665707

[B2] American College of Obstetricians and Gynecologists, and Task Force on Hypertension in Pregnancy (2013). Hypertension in pregnancy. Report of the american college of obstetricians and gynecologists’ task force on hypertension in pregnancy. *Obstet Gynecol.* 122 1122–1131. 24150027 10.1097/01.AOG.0000437382.03963.88

[B3] Ashton S. V. Whitley G. S. Dash P. R. Wareing M. Crocker I. P. Baker P. N. (2005). Uterine spiral artery remodeling involves endothelial apoptosis induced by extravillous trophoblasts through Fas/FasL interactions. *Arterioscler. Thromb. Vasc. Biol.* 25 102–108. 10.1161/01.atv.0000148547.70187.89 15499040 PMC4228192

[B4] Baker C. D. Ryan S. L. Ingram D. A. Seedorf G. J. Abman S. H. Balasubramaniam V. (2009). Endothelial colony-forming cells from preterm infants are increased and more susceptible to hyperoxia. *Am. J. Respir. Crit. Care Med.* 180 454–461. 10.1164/rccm.200901-0115OC 19483112 PMC2742761

[B5] Bamberger A. M. Schulte H. M. Thuneke I. Erdmann I. Bamberger C. M. Asa S. L. (1997). Expression of the apoptosis-inducing Fas ligand (FasL) in human first and third trimester placenta and choriocarcinoma cells. *J. Clin. Endocrinol. Metab.* 82 3173–3175. 10.1210/jcem.82.9.4360 9284765

[B6] Ben Ali Gannoun M. Bourrelly S. Raguema N. Zitouni H. Nouvellon E. Maleh W. (2016). Placental growth factor and vascular endothelial growth factor serum levels in tunisian arab women with suspected preeclampsia. *Cytokine* 79 1–6. 10.1016/j.cyto.2015.12.005 26702929

[B7] Burton G. J. Fowden A. L. (2015). The placenta: a multifaceted, transient organ. *Philos. Trans. R. Soc. Lond. B Biol. Sci.* 370:20140066. 10.1098/rstb.2014.0066 25602070 PMC4305167

[B8] Chaiworapongsa T. Chaemsaithong P. Yeo L. Romero R. (2014). Pre-eclampsia part 1: current understanding of its pathophysiology. *Nat. Rev. Nephrol.* 10 466–480. 10.1038/nrneph.2014.102 25003615 PMC5893150

[B9] Chen L. J. Han Z. Q. Zhou H. Zou L. Zou P. (2010). Inhibition of HLA-G expression via RNAi abolishes resistance of extravillous trophoblast cell line TEV-1 to NK lysis. *Placenta* 31 519–527. 10.1016/j.placenta.2010.03.008 20430441

[B10] Coleman S. J. Gerza L. Jones C. J. Sibley C. P. Aplin J. D. Heazell A. E. (2013). Syncytial nuclear aggregates in normal placenta show increased nuclear condensation, but apoptosis and cytoskeletal redistribution are uncommon. *Placenta* 34 449–455. 10.1016/j.placenta.2013.02.007 23507147 PMC3661987

[B11] Cornelius D. C. (2018). Preeclampsia: from inflammation to immunoregulation. *Clin. Med. Insights Blood Disord.* 11:1179545x17752325. 10.1177/1179545X17752325 29371787 PMC5772493

[B12] da Cunha Castro E. C. Popek E. (2018). Abnormalities of placenta implantation. *APMIS* 126 613–620. 10.1111/apm.12831 30129132

[B13] de Oliveira L. G. Karumanchi A. Sass N. (2010). Preeclampsia: oxidative stress, inflammation and endothelial dysfunction. *Revist. Bras. Ginecol. Obstetr.* 32 609–616. 10.1590/s0100-72032010001200008 21484030

[B14] Dong X. Gou W. Li C. Wu M. Han Z. Li X. (2017). Proteinuria in preeclampsia: not essential to diagnosis but related to disease severity and fetal outcomes. *Pregnancy Hypertens.* 8 60–64. 10.1016/j.preghy.2017.03.005 28501282

[B15] Duhig K. Vandermolen B. Shennan A. (2018). Recent advances in the diagnosis and management of pre-eclampsia. *F1000Research* 7:242. 10.12688/f1000research.12249.1 29560262 PMC5832913

[B16] Dymara-Konopka W. Laskowska M. Blazewicz A. (2018). Angiogenic imbalance as a contributor of preeclampsia. *Curr. Pharm. Biotechnol.* 19 797–815. 10.2174/1389201019666180925115559 30255753

[B17] Eddy A. C. Bidwell G. L. III George E. M. (2018). Pro-angiogenic therapeutics for preeclampsia. *Biol. Sex Differ.* 9:36. 10.1186/s13293-018-0195-5 30144822 PMC6109337

[B18] Elmore S. (2007). Apoptosis: a review of programmed cell death. *Toxicol. Pathol.* 35 495–516. 17562483 10.1080/01926230701320337 PMC2117903

[B19] Faas M. M. de Vos P. (2017a). Maternal monocytes in pregnancy and preeclampsia in humans and in rats. *J. Reprod. Immunol.* 119 91–97. 10.1016/j.jri.2016.06.009 27396500

[B20] Faas M. M. de Vos P. (2017b). Uterine NK cells and macrophages in pregnancy. *Placenta* 56 44–52. 10.1016/j.placenta.2017.03.001 28284455

[B21] Figueiredo A. S. Schumacher A. (2016). The T helper type 17/regulatory T cell paradigm in pregnancy. *Immunology* 148 13–21. 10.1111/imm.12595 26855005 PMC4819144

[B22] Fogarty N. M. Ferguson-Smith A. C. Burton G. J. (2013). Syncytial knots (Tenney-Parker changes) in the human placenta: evidence of loss of transcriptional activity and oxidative damage. *Am. J. Pathol.* 183 144–152. 10.1016/j.ajpath.2013.03.016 23680657

[B23] Harris S. L. Levine A. J. (2005). The p53 pathway: positive and negative feedback loops. *Oncogene* 24 2899–2908. 10.1038/sj.onc.1208615 15838523

[B24] Heazell A. E. Moll S. J. Jones C. J. Baker P. N. Crocker I. P. (2007). Formation of syncytial knots is increased by hyperoxia, hypoxia and reactive oxygen species. *Placenta* 28(Suppl. A), S33–S40. 17140657 10.1016/j.placenta.2006.10.007

[B25] Heerema-McKenney A. (2018). Defense and infection of the human placenta. *APMIS* 126 570–588. 10.1111/apm.12847 30129129

[B26] Helmo F. R. Lopes A. M. M. Carneiro A. Campos C. G. Silva P. B. Dos Reis Monteiro M. L. G. (2018). Angiogenic and antiangiogenic factors in preeclampsia. *Pathol. Res. Pract.* 214 7–14. 10.1016/j.prp.2017.10.021 29174227

[B27] Hung T. H. Skepper J. N. Charnock-Jones D. S. Burton G. J. (2002). Hypoxia-reoxygenation: a potent inducer of apoptotic changes in the human placenta and possible etiological factor in preeclampsia. *Circ. Res.* 90 1274–1281. 10.1161/01.res.0000024411.22110.aa 12089065

[B28] Hutabarat M. Wibowo N. Huppertz B. (2017). The trophoblast survival capacity in preeclampsia. *PLoS One* 12:e0186909. 10.1371/journal.pone.0186909 29107968 PMC5673174

[B29] Jafri S. Ormiston M. L. (2017). Immune regulation of systemic hypertension, pulmonary arterial hypertension, and preeclampsia: shared disease mechanisms and translational opportunities. *Am. J. Physiol. Regul. Integr. Comp. Physiol.* 313 R693–R705. 10.1152/ajpregu.00259.2017 28978513

[B30] Karthikeyan V. J. Lip G. Y. (2011). Endothelial damage/dysfunction and hypertension in pregnancy. *Front. Biosci.* 3 1100–1108. 10.2741/e314 21622117

[B31] Karthikeyan V. J. Lip G. Y. Lane D. A. Blann A. D. (2011). Angiogenin and apoptosis in hypertension in pregnancy. *Pregnancy Hypertens.* 1 191–196. 10.1016/j.preghy.2011.07.002 26009025

[B32] Kauma S. W. Huff T. F. Hayes N. Nilkaeo A. (1999). Placental Fas ligand expression is a mechanism for maternal immune tolerance to the fetus. *J. Clin. Endocrinol. Metab.* 84 2188–2194. 10.1210/jc.84.6.2188 10372730

[B33] Kestlerova A. Feyereisl J. Frisova V. Mechurova A. Sula K. Zima T. (2012). Immunological and biochemical markers in preeclampsia. *J. Reprod. Immunol.* 96 90–94. 10.1016/j.jri.2012.10.002 23131770

[B34] Knofler M. Haider S. Saleh L. Pollheimer J. Gamage T. James J. (2019). Human placenta and trophoblast development: key molecular mechanisms and model systems. *Cell. Mol. Life Sci.* 76 3479–3496. 10.1007/s00018-019-03104-6 31049600 PMC6697717

[B35] Liu H. Liu X. Jin H. Yang F. Gu W. Li X. (2013). Proteomic analysis of knock-down HLA-G in invasion of human trophoblast cell line JEG-3. *Int. J. Clin. Exp. Pathol.* 6 2451–2459. 24228107 PMC3816814

[B36] Liu S. Diao L. Huang C. Li Y. Zeng Y. Kwak-Kim J. Y. H. (2017). The role of decidual immune cells on human pregnancy. *J. Reprod. Immunol.* 124 44–53. 10.1016/j.jri.2017.10.045 29055791

[B37] Loukeris K. Sela R. Baergen R. N. (2010). Syncytial knots as a reflection of placental maturity: reference values for 20 to 40 weeks’ gestational age. *Pediatr. Dev. Pathol.* 13 305–309. 10.2350/09-08-0692-OA.1 20017638

[B38] Matthiesen L. Berg G. Ernerudh J. Ekerfelt C. Jonsson Y. Sharma S. (2005). Immunology of preeclampsia. *Chem. Immunol. Allergy* 89 49–61. 10.1159/000087912 16129952

[B39] Mayrink J. Costa M. L. Cecatti J. G. (2018). Preeclampsia in 2018: revisiting concepts, physiopathology, and prediction. *Sci. World J.* 2018:6268276. 10.1155/2018/6268276 30622442 PMC6304478

[B40] Miko E. Szereday L. Barakonyi A. Jarkovich A. Varga P. Szekeres-Bartho J. (2009). Immunoactivation in preeclampsia: Vdelta2+ and regulatory T cells during the inflammatory stage of disease. *J. Reprod. Immunol.* 80 100–108. 10.1016/j.jri.2009.01.003 19395088

[B41] Moffett-King A. (2002). Natural killer cells and pregnancy. *Nat. Rev. Immunol.* 2 656–663. 12209134 10.1038/nri886

[B42] Osol G. Mandala M. (2009). Maternal uterine vascular remodeling during pregnancy. *Physiology* 24 58–71. 10.1152/physiol.00033.2008 19196652 PMC2760472

[B43] Pongcharoen S. Searle R. F. Bulmer J. N. (2004). Placental Fas and Fas ligand expression in normal early, term and molar pregnancy. *Placenta* 25 321–330. 10.1016/j.placenta.2003.08.020 15028424

[B44] Racicot K. Kwon J. Y. Aldo P. Silasi M. Mor G. (2014). Understanding the complexity of the immune system during pregnancy. *Am. J. Reprod. Immunol.* 72 107–116. 10.1111/aji.12289 24995526 PMC6800182

[B45] Raguema N. Zitouni H. Ben Ali Gannoun M. Benletaifa D. Almawi W. Y. Mahjoub T. (2018). FAS A-670G and Fas ligand IVS2nt A 124G polymorphisms are significantly increased in women with pre-eclampsia and may contribute to HELLP syndrome: a case-controlled study. *BJOG* 125 1758–1764. 10.1111/1471-0528.15412 30066360

[B46] Rajakumar A. Cerdeira A. S. Rana S. Zsengeller Z. Edmunds L. Jeyabalan A. (2012). Transcriptionally active syncytial aggregates in the maternal circulation may contribute to circulating soluble fms-like tyrosine kinase 1 in preeclampsia. *Hypertension* 59 256–264. 10.1161/HYPERTENSIONAHA.111.182170 22215706 PMC3319764

[B47] Redman C. W. Sargent I. L. (2005). Latest advances in understanding preeclampsia. *Science* 308 1592–1594. 10.1126/science.1111726 15947178

[B48] Roberts J. M. Hubel C. A. (2009). The two stage model of preeclampsia: variations on the theme. *Placenta* 30(Suppl. A), S32–S37. 10.1016/j.placenta.2008.11.009 19070896 PMC2680383

[B49] Robillard P. Y. Dekker G. Chaouat G. Hulsey T. C. Saftlas A. (2011). Epidemiological studies on primipaternity and immunology in preeclampsia–a statement after twelve years of workshops. *J. Reprod. Immunol.* 89 104–117. 10.1016/j.jri.2011.02.003 21543120

[B50] Roh C. R. Lee J. W. Kang B. H. Yang S. H. Kim B. G. Bae D. S. (2002). Differential expressions of Fas and Fas ligand in human placenta. *J. Korean Med. Sci.* 17 213–216. 11961305 10.3346/jkms.2002.17.2.213 PMC3054862

[B51] Salamonsen L. A. Dimitriadis E. Jones R. L. Nie G. (2003). Complex regulation of decidualization: a role for cytokines and proteases–a review. *Placenta* 24(Suppl. A), S76–S85. 12842418 10.1053/plac.2002.0928

[B52] Schoots M. H. Gordijn S. J. Scherjon S. A. van Goor H. Hillebrands J. L. (2018). Oxidative stress in placental pathology. *Placenta* 69 153–161. 10.1016/j.placenta.2018.03.003 29622278

[B53] Sharp A. N. Heazell A. E. Baczyk D. Dunk C. E. Lacey H. A. Jones C. J. (2014). Preeclampsia is associated with alterations in the p53-pathway in villous trophoblast. *PLoS One* 9:e87621. 10.1371/journal.pone.0087621 24498154 PMC3907567

[B54] Sharp A. N. Heazell A. E. Crocker I. P. Mor G. (2010). Placental apoptosis in health and disease. *Am. J. Reprod. Immunol.* 64 159–169. 10.1111/j.1600-0897.2010.00837.x 20367628 PMC3025811

[B55] Taylor E. B. Sasser J. M. (2017). Natural killer cells and T lymphocytes in pregnancy and pre-eclampsia. *Clin. Sci.* 131 2911–2917. 10.1042/CS20171070 29222389

[B56] Thellin O. Heinen E. (2003). Pregnancy and the immune system: between tolerance and rejection. *Toxicology* 185 179–184. 10.1016/s0300-483x(02)00607-8 12581692

[B57] Tomas S. Z. Prusac I. K. Roje D. Tadin I. (2011). Trophoblast apoptosis in placentas from pregnancies complicated by preeclampsia. *Gynecol. Obstet. Invest.* 71 250–255. 10.1159/000320289 21266791

[B58] Tsatsaris V. Fournier T. Winer N. (2008). Pathophysiology of preeclampsia. *J. Gynecol. Obstet. Biol. Reprod.* 37 16–23. 10.1016/j.jgyn.2007.08.003 18036745

[B59] Whitley G. S. Cartwright J. E. (2009). Trophoblast-mediated spiral artery remodelling: a role for apoptosis. *J. Anat.* 215 21–26. 10.1111/j.1469-7580.2008.01039.x 19215319 PMC2714635

[B60] Whitley G. S. Dash P. R. Ayling L. J. Prefumo F. Thilaganathan B. Cartwright J. E. (2007). Increased apoptosis in first trimester extravillous trophoblasts from pregnancies at higher risk of developing preeclampsia. *Am. J. Pathol.* 170 1903–1909. 10.2353/ajpath.2007.070006 17525258 PMC1899436

[B61] Yie S. M. Li L. H. Li Y. M. Librach C. (2004). HLA-G protein concentrations in maternal serum and placental tissue are decreased in preeclampsia. *Am. J. Obstet. Gynecol.* 191 525–529. 10.1016/j.ajog.2004.01.033 15343231

